# Internal Gene Cassette From a Human-Origin H7N9 Influenza Virus Promotes the Pathogenicity of H9N2 Avian Influenza Virus in Mice

**DOI:** 10.3389/fmicb.2020.01441

**Published:** 2020-07-22

**Authors:** Miaomiao Zhang, Chen Zhao, Hongjun Chen, Qiaoyang Teng, Lang Jiang, Daobin Feng, Xuesong Li, Songhua Yuan, Jianqing Xu, Xiaoyan Zhang, Zejun Li

**Affiliations:** ^1^Shanghai Veterinary Research Institute, Chinese Academic of Agricultural Sciences & Animal Influenza Virus Evolution and Pathogenesis Innovation Team of the Agricultural Science and Technology Innovation Team, Shanghai, China; ^2^Scientific Research Center, Shanghai Public Health Clinical Center & Institutes of Biomedical Sciences, Key Laboratory of Medical Molecular Virology of Ministry of Education/Health, Shanghai Medical College, Fudan University, Shanghai, China

**Keywords:** avian influenza, H9N2, H7N9, internal gene, pathogenicity

## Abstract

H9N2 avian influenza virus is one of the most widely circulating viruses in poultry and poses a huge potential threat to human health due to its frequent gene reassortment with other influenza viruses. In this study, we generated a series of H9N2-H7N9 reassortant viruses and examined their pathogenicity in a mouse model. We found that HA or combined HA and NA replacement on the H9N2 background led to no substantial change in the virus-induced pathogenicity, whereas H9N2 virus containing H7N9 internal genes had significantly higher virulence in comparison to the parental H9N2 virus. This increased pathogenicity is associated with enhanced viral replication both in mice and in MDCK cells. We further demonstrated that the viral ribonucleoprotein complex from H7N9 virus possessed higher activity than that from its H9N2 counterpart. Collectively, our data demonstrated that genetic compatibility between H9N2 and H7N9 viruses facilitated the reassortment between H7N9 and H9N2 viruses co-circulated in poultry and that internal gene replacement would convert H9N2 virus into a novel threat to human health.

## Introduction

H9N2 avian influenza virus (AIV) is the most widely circulating AIV in poultry populations globally. H9N2 viruses were first isolated from turkeys in American in 1966 (Homme and Easterday, 1970). In the early 1990s, H9N2 virus became endemic in poultry across most of Asia, the Middle East, and North and West Africa (Guan et al., 1999). It is noteworthy that H9N2 AIV has already acquired the ability to cross the species barrier and infect humans without adaptation in intermediate hosts. An early H9N2 human infection cases in China could be traced back to two cases of child infection reported in Hong Kong in 1998 and 1999, respectively (Guo et al., 1999; Peiris et al., 1999). Subsequent human infections were confirmed in several countries, including Egypt, Pakistan, Bangladesh, and Oman (Ali et al., 2019; Peacock et al., 2019). Recently, the reported incidence of H9N2 infection has significantly increased in China, most likely owing to the ongoing screening for zoonotic H7N9 infections (Peacock et al., 2019). To date, approximately 60 human H9N2 infections have been confirmed in the laboratory, and most of those cases had a history of exposure to poultry^1^. Moreover, viral genome analysis revealed that HA genes of the human H9N2 isolates belonged to the G1-W, G1-E, or BJ94 lineages and were related closely to the viruses isolated from local poultry (Peiris et al., 1999; Huang et al., 2015).

H9N2 viruses are often found to be co-circulated among poultry with other AIV subtypes, increasing the rates of reassortment between these viruses and thus posing a huge threat to public health. H9N2 AIV could serve as gene donor to supply internal genes to other viruses, including the H7N9, H10N8, and H5N6 influenza viruses that recently caused human infection (Liu et al., 2013; Chen et al., 2014; Yang et al., 2015). Reciprocally, H9N2 viruses continually obtain single or multiple genes from other AIVs, exampled by the evolutionary trajectory of predominant H9N2 viruses circulating in Pakistan and Bangladesh, the viruses are boosted by exchange of genes with H7N3 and H5N1 HPAIV (Iqbal et al., 2009; Parvin et al., 2014; Shanmuganatham et al., 2014). Multiple H9N2 genotypes containing the internal genes from H5N1 HPAIV were found in China (Dong et al., 2011).

H9N2 infections, distinct from H7N9 infections, normally only cause mild symptoms in human, and therefore, the potential risk of enhanced virulence and/or transmissibility resulting from reassortment with other viruses is easy ignore. In this study, we aimed to address the risk that H7N9 human influenza virus will provide internal genes to H9N2 AIV.

## Materials and Methods

### Viruses

A/Chicken/Shanghai/F/98 (CK/F98) H9N2 virus (Genbank accession number: AY253750.1-AY253756.1, AY743216.1) isolated from chicken in China was conserved at our laboratory. A/Shanghai/4664T/2013(SH/4664) H7N9 virus (Genbank accession number: KC853225.1-KC853232.1) was conserved at a Biosafety Level 3 (BSL3) lab at Shanghai Public Clinical Center. For the generation of recombinant virus by reverse genetics, the cDNA of genes from CK/F98 or SH/4664 was PCR-amplified and inserted into pHW2000 vector through *Bsm*BI cutting sites. Viruses were rescued by using an eight-plasmid reverse genetics system Pusch and Suarez, 2018. Three H9N2-H7N9 hybrid viruses were thus generated with HA genes, or HA and NA genes, or the six internal genes derived from H7N9 and other genes from H9N2, designated CK/F98-4664 HA, CK/F98-4664 HA/NA, and CK/F98-4664int, respectively. As the control, the parental H9N2 virus was also rescued by reverse genetics following the same experimental procedure. Rescued viruses were propagated in 9-day-old SPF chicken embryos (Boehringer-Ingelheim Co., Beijing) and confirmed by sequencing. The titers of virus stocks were determined as 50% egg infectious dose (EID50) or 50% tissue culture infectious dose (TCID50) in accordance with the Reed–Muench method.

### Animal Studies

Six-week-old female BALB/c mice (Charles River Co., Beijing, China) were maintained under specific pathogen-free conditions at the animal facilities of Shanghai Public Health Clinical Center. All animal studies were performed in strict accordance with the Home Office regulations and were approved by the Shanghai Public Health Center Local Ethical Committee. The experiments involving live viruses were carried out in a BSL3 facility at Shanghai Public Clinical Center conforming to the institutional biosafety manual. For virus challenge, mice anesthetized with 5% pentobarbital were intranasally inoculated with a dose of 1 × 10^6^EID_50_ per 50 μl virus (eight mice per group). Additionally, five mice were inoculated with 50 μl PBS buffer, serving as negative controls. Among the experimental group, five mice were monitored daily for 14 days post-inoculation (dpi) for body weight and mortality. Mice with 30% body weight loss or more were considered dead and were euthanized. The other three mice were sacrificed at 4 dpi to determine virus titers in the lung, nasal turbinate, liver, spleen, kidney, and brain. For virus titration, tissues were weighed and homogenized using the TissueLyser II (Qiagen) to produce a concentration of 0.1 g/ml (wt/vol) homogenate in 0.01 M PBS. After centrifugation at 12,000 rpm for 10 min, 100-μl aliquots of the supernatants were collected, and a series of dilutions were made for inoculating 9-day-old embryonated eggs. Virus titers were subsequently determined by the Reed–Muench method.

### Minigenome Assay

A minigenome assay was used to measure the activity of reconstituted viral ribonucleoprotein (vRNP), as reported previously (Bortz et al., 2011). Briefly, the PA, PB1, PB2, and NP genes derived from CK/F98 and SH/4664 viruses were individually cloned into pCAGGS expression vector. Transfection into 293T cells was performed with three technical replicates using Mirus transfection reagent in a 24-well plate. The cells in each well were co-transfected with 100 ng of each of the pCAGGS plasmids expressing PA, PB1, PB2, and NP, 100 ng of a reporter plasmid carrying Firefly luciferase in negative sense flanked by 5′ and 3′ terminal ends of the influenza NS, and 20 ng of control reporter plasmid with Renilla luciferase derived by a thymidine kinase promoter for constitutive expression. The cells were collected at 24 h post-transfection and subjected to testing of the activities of luciferases using a dual-luciferase reporter assay system (Promega) on a GloMax 96 microplate luminometer (Promega). The activity of vRNP polymerase was indicated as the ratio of the Firefly to the Renilla fluorescence value.

### Viral Replication on MDCK Cells

Confluent MDCK cells in a 25-cm flask were infected with the indicated viruses at a multiplicity of infection (MOI) of 0.001. The viral inoculums were removed after 1 h of adsorption at 37°C and washed three times with PBS, followed by the addition of serum-free Dulbecco’s Modified Eagle Medium supplemented with 1 μg/ml trypsin treated with L-(tosylamido-2-phenyl) ethyl chloromethyl ketone (TPCK). Infected cells were further incubated at 37°C, 5% CO_2_ in a humidified incubator. Aliquots of cell-culture supernatants were collected at indicated time points post-infection and used for virus titration in MDCK cells.

### Statistical Analyses

All of the statistical analyses were performed using Prism 7 software (Graphpad, La Jolla, CA). Data were expressed as mean ± SD, except for analysis of mouse body weight change, where mean ± SEM was used. For comparison between two groups, statistical significance was assessed using a two-tailed Student’s *t*-test. When more than two groups were compared, one-way ANOVA was applied in combination with the *post hoc* Bonferroni test.

## Results

### H9N2 Hybird Virus Containing HA Gene From H7N9 Virus Showed Low Virulence in Mice

**FIGURE 1:**
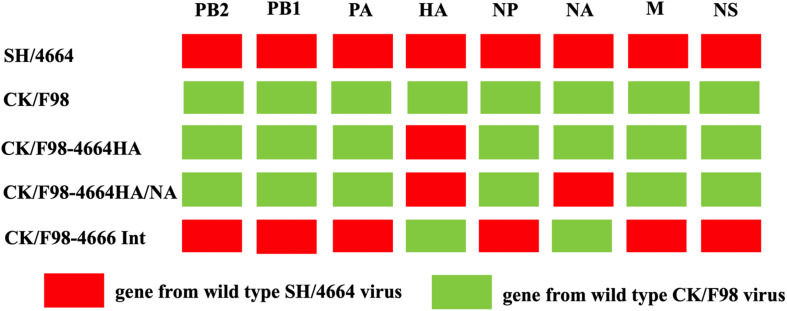
Schematic of H7N9-H9N2 hybrid viruses analyzed in this study.

**FIGURE 2:**
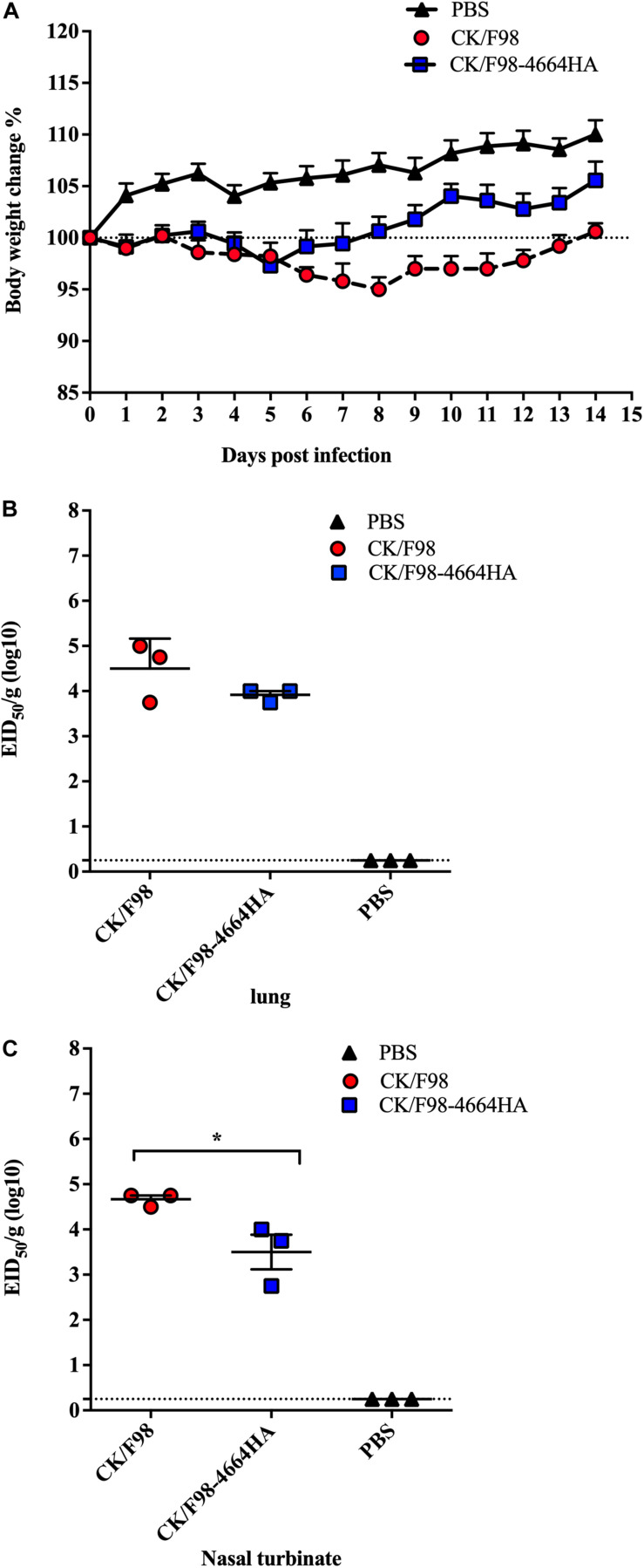
H7N9-derived HA was not able to enhance the pathogenicity and replication of H9N2 virus in mouse. Mice were intranasally inoculated with a single dose of 10^6^ EID_50_ of either the parental H9N2 CK/F98 virus or the CK/F98-4664HA hybrid virus with HA gene being replaced by its counterpart from H7N9 SH/4664 virus. **(A)** Weight loss during a 14-day observation period. **(B)** Virus titers in animal lungs, as determined by EID50 using MDCK cells. **(C)** Virus titers in animal nasal turbinates, as determined by EID50 using MDCK cells. *n* = 3 for each group. Data are expressed as mean ± SEM. *P < 0.05 by one-way ANOVA.

We selected two viruses identified in China, namely, H9N2 avian strain CK/F98 and H7N9 SH/4664 strain, to study the effect of genetic reassortment with human H7N9 on the replication and pathogenicity of avian H9N2 using a mouse infection model (Figure 1). We first focused on the effect of HA by replacing the HA gene of the H9N2 virus with that of the H7N9 virus. The recombinant virus thus generated, namely, CK/F98-4664HA, along with the parental CK/F98 virus were used to individually infect mice by intranasal inoculation at a dose of 10^6^ EID_50_. All of the infected mice survived up to 14 days after virus challenge. Both viruses caused a slight weight decrease in the infected mice in the early period that lasted for 5 and 8 days for CK/F98-4664HA and CK/F98 virus, respectively. After that, the weight of the mice in both groups start to increase. CK/F98-4664HA infection had weaker weight effects than the parental virus CK/F98, including a higher nadir and faster rebound. Accordingly, compared to the weight before infection, the former group attained an average weight of approximately 5% higher at 14 dpi, whereas the CK/F98 group showed essentially no gain (Figure 2A). At 4 dpi, average virus titer in the lung of the CK/F98-4664 HA group was 4.6810^3^ EID_50_/g, which was slightly lower than that of the CK/F98 H9N2 group, whose average virus titer was 1.7810^4^EID_50_/g (Figure 2B). The same virus titer trend was observed in the nasal turbinate, with 1.78 × 10^3^EID_50_/g for the CK/F98-4664 HA group versus 2.63 × 10^4^EID_50_/g for the CK/F98 H9N2 group (*P* < 0.05; Figure 2C). Viruses were undetectable in the brain, spleen, liver, and kidney of the mice in both groups. Collectively, these results demonstrated that the HA gene of H7N9 virus alone cannot promote the virulence of reassortant virus in mice.

### H9N2 Hybrid Virus With HA and NA Genes From H7N9 Virus Showed Moderate Virulence in Mice

**FIGURE 3:**
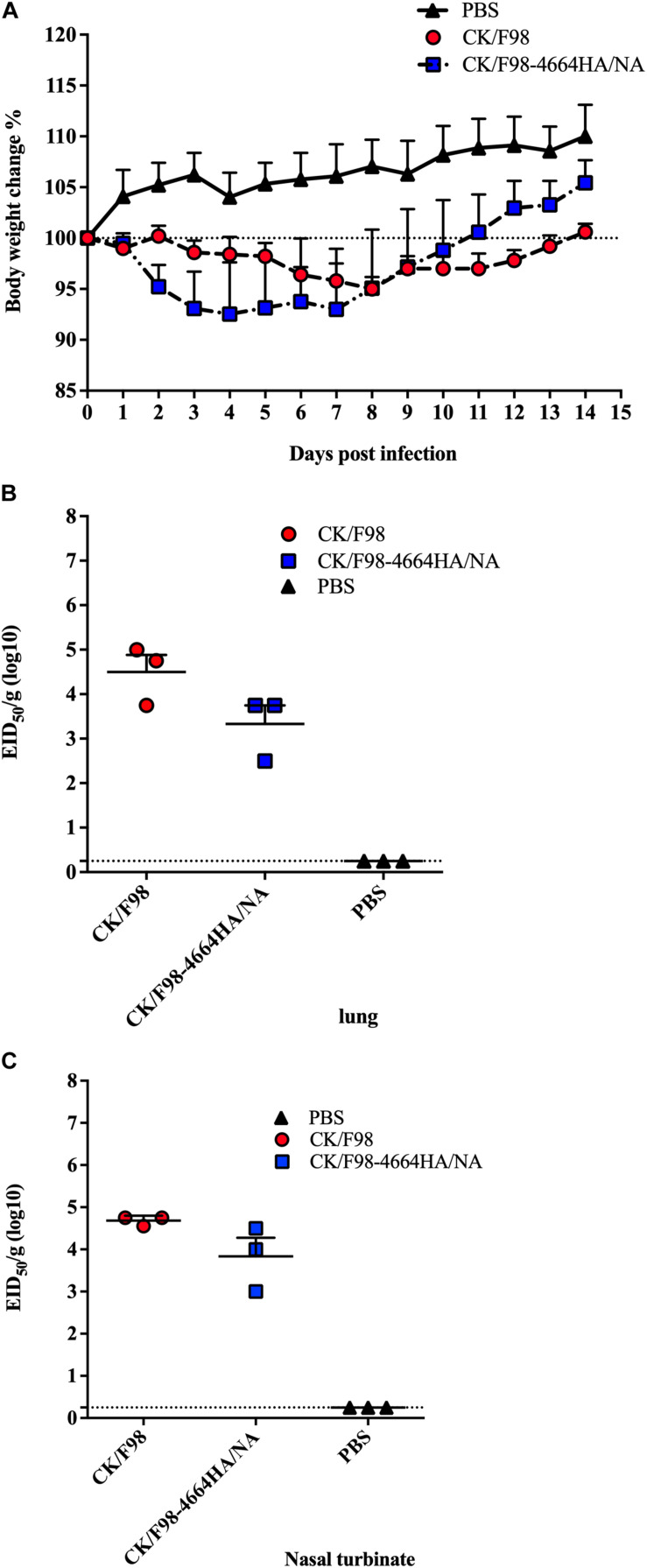
H7N9 surface genes were not sufficient to convert low pathogenicity and replication of H9N2 virus in mouse. Mice were intranasally inoculated with a single dose of 10^6^ EID50 of either the parental H9N2 CK/F98 virus or the CK/F98-4664HA/NA hybrid virus with both HA and NA genes derived from H7N9 SH/4664 virus. **(A)** Weight loss during a 14-day observation period. **(B)** Virus titers in animal lungs, as determined by EID50 using MDCK cells. **(C)** Virus titers in animal nasal turbinates, as determined by EID50 using MDCK cells. *n* = 3 for each group. Data are expressed as mean ± SEM. Statistical significance was analyzed by one-way ANOVA.

Considering the importance of HA/NA balance in virus pathogenicity, we next generated the reassortant virus CK/F98-4664HA/NA with the HA and NA genes derived from SH/4664 (H7N9) virus on the background of H9N2 virus. To evaluate the pathogenecity of that virus, mice were intranasally infected, as described above. All of the CK/F98-4664HA/NA-infected mice survived at 14 dpi. As compared to the CF/F98 group, the CK/F98-4664HA/NA group displayed more rapid weight loss in the early stage, reaching the nadir at 4 dpi (Figure 3A). In addition, the weight nadir of the CK/F98-4664HA/NA group was slightly lower than that of the CK/F98 group (Figure 3A). However, the former recovered faster than the latter, resulting in more weight gain at 14 dpi relative to the initial weight at the time of virus challenge (Figure 3A). The virus titers of CK/F98-4664HA/NA in the lungs and nasal turbinates were similar to those of CK/F98 virus (Figures 3B,C). These results excluded surface genes as the primary virulence determinants distinguishing H7N9 from H9N2 in mouse infection.

### The Internal Gene Constellation of H7N9 Virus Conferred H9N2 Increased Pathogenicity in Mice

**FIGURE 4:**
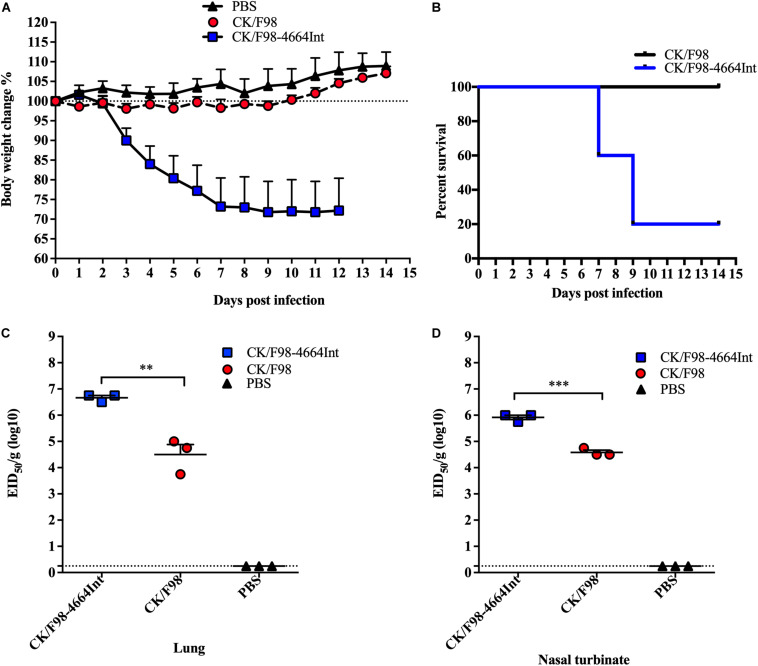
Acquisition of the internal gene constellation of H7N9 virus resulted in enhanced pathogenicity and replication of H9N2 virus in mouse. Mice were intranasally inoculated with a single dose of 10^6^ EID_50_ of the parental H9N2 CK/F98 virus or the CK/F98-4664Int virus with the six internal genes derived from H7N9 SH/4664 virus. **(A)** Weight loss during a 14-day observation period. **(B)** Survival curve during a 14-day-observation period. Mice that lost >25% of their pre-inoculated weight were regarded as dead and euthanized. **(C)** Virus titers in animal lungs, as determined by EID50 using MDCK cells. **(D)** Virus titers in animal nasal turbinates, as determined by EID50 using MDCK cells. *n* = 3 for each group. Data are expressed as mean ± SEM. *P* < 0.05, **P < 0.01, and ***P < 0.001 by one-way ANOVA.

Next, we aimed to replace the internal gene constellation of CK/F98 by that of SH/4664 (H7N9) by reverse genetics. This reassortant virus, designated as CK/F98-4664Int, was successfully generated and subsequently examined in a mouse infection model. Unlike the surface gene replacement viruses and parental virus CK/F98, CK/F98-4664Int caused drastic weight loss, reaching a nadir of 28% weight loss within 9 dpi (Figure 4A). There was only 20% survival of mice infected with CK/F98-4664int at 14 dpi, whereas 100% of mice infected with CK/F98 survived (Figure 4B). The enhanced virulence of CK/F98-4664Int virus was also reflected in virus replication in mice. At 4 dpi, the average virus titer in the lungs of CK/F98-4664int-infected mice was 4.6810^6^ EID_50_/g, which was significantly higher than that of CK/F98-infected mice (*P* < 0.01;

Figure 4C). The virus titers in nasal turbinates showed a similar trend, being 22-fold higher than that of CK/F98-infected mice (*P* < 0.001; Figure 4D). There was no detectable virus titer for either virus in other tissues, including liver, spleen, kidney, and brain. The results indicated that the internal genes from H7N9 virus promoted the replication and pathogenicity of H9N2 virus in mice.

### H9N2 Virus With Internal Genes of H7N9 Virus Replicated More Effectively in Mammalian Cells, Likely Due to Enhanced Polymerase Activity

**FIGURE 5:**
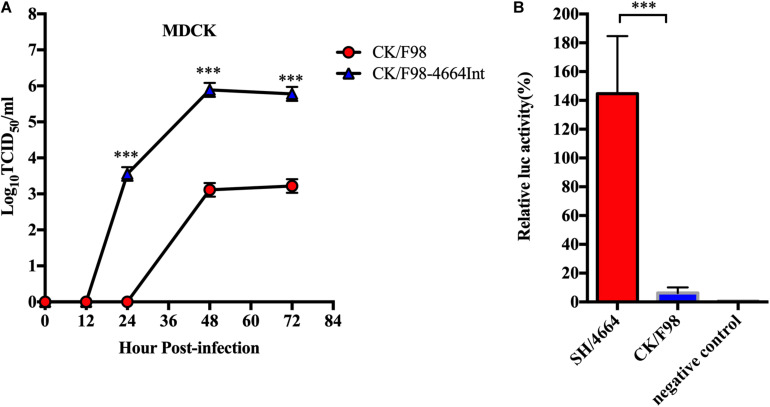
Cell culture studies validated the superiority of H7N9 over H9N2 in their internal genes, likely the viral ribonucleoprotein complex, to support viral replication in mammal cells. **(A)** Multiple growth curves of CK/98 and CK/F98-4664Int virus in MDCK cells. MOI = 0.001. Virus titers in the supernatant at the indicated time points were determined by TCID50 using MDCK cells. Data represent one of three independent experiments, expressed as mean ± SEM. **(B)** Analyses of vRNP activity by minigenome assay. A set of four plasmids individually expressing PA1, PB1, PB2, and NP of CK/98 or SH/4664 was co-transfected into 293T cells with: (1) a reporter construct containing negative-sense Firefly luciferase gene flanked by the 5’ and 3’ ends of IAV NS1 gene; (2) a control reporter gene constitutively expressing Renilla luciferase. After 48 h, transfected cells were harvested, and vRNP activities were measured as Firefly:Renilla luciferase ratios. Error bars represent standard error from three biological replicates. ***P < 0.001, one way-ANOVA.

We further analyzed the kinetics of replication of CK/F98-4664int versus parental CK/F98 on MDCK cells. MDCK cells were infected with each of the two viruses at an MOI of 0.001, and the virus titers in the supernatant were tested on MDCK cells at different numbers of hours post-infection. CK/F98-4664Int proliferated significantly faster than CK/F98, and the titer reached 3.5 log_10_ TCID_50_ in the supernatant at 24 hours post inoculation (h.p.i.), whereas there was no virus detectable in the supernatant of cells infected with the latter. The virus titer of CK/F98-4664Int-infected cells was 100–1000 times higher than that of CK/F98 infected cells at 48 and 72 h.p.i. (*P* < 0.001; Figure 5A). These results suggested that internal genes of H7N9 supported efficient replication of H9N2 virus on mammalian cells.

To test the activities of vRNPs, minigenomes derived from H9N2 and H7N9 viruses were analyzed on 293T cells. The relative activity of vRNPs from H7N9 virus was about 7-fold higher than that of vRNPs from H9N2 virus (*P* < 0.001; Figure 5B). Thus, the superiority of H7N9 virus over H9N2 virus in replication in mammalian cells might stem from an intrinsically more active vRNP or a vRNP that is more adaptive to mammalian hosts.

## Discussion

As one of most widely circulating influenza viruses in chickens (Pusch and Suarez, 2018), H9N2 is commonly viewed as a low pathogenicity virus causing mild or no symptoms. Thus, their threats to human health have been considered negligible as compared to the notorious H7 or H5 strains. In this study, utilizing recombinant virus generated by reverse genetics, we discovered that the capability of an avian H9N2 virus to infect and replicate in mice could be remarkably enhanced by obtaining an internal gene constellation from an H7N9 virus that has been transmitted to human since 2013 and has caused hundreds of human deaths to date. This would suggest reassortment between avian H9N2 and human H7N9 during co-infection as a possible natural mechanism to yield novel H9N2 virus with increased replication capacity in mammals.

Previous studies have suggested that H9N2 AIVs can act as either donors or recipients of genes when co-circulated with other virus strains (Dong et al., 2011; Liu et al., 2013; Chen et al., 2014; Yang et al., 2015). Here, we have focused on their property changes in receipt of genes from H7N9 viruses. Given the important role of HA in the restriction of the host range and spreading capability of a virus, we first generated a recombinant H9N2 virus in which the cognate HA gene was replaced by its H7N9 counterpart. However, such replacement did not result in increased pathogenicity and virus titer. This result indicated that the H9N2 strain being studied, despite its avian origin, has obtained the capability to bind and enter mammal cells. It should also be noted that, although H7N9 is classified as highly pathogenic avian influenza virus (HPAIV) because its HA can evolve to possess a multi-basic cleavage site for systemic spread, the H7N9 strain and the H9N2 counterpart used in our study both featured a mono-basic cleavage site in HA protein that limits virus replication within respiratory tissues (Nao et al., 2017).

Our animal studies revealed that the *in vivo* pathogenicity was highly correlated with virus titers. Thus, the CK/F98-4664Int virus with internal genes entirely from the H7N9 strain combined with a couple of H9N2 surface genes yielded significantly higher viral titer in lung and nasal turbinate as compared to H9N2 CK/F98, in line with substantially increased mortality. On the contrary, either the CK/F98-4664HA or CK/F98-4664HA/NA virus, with HA or both HA and NA from H7N9 virus and retaining the CK/F98 internal gene constellation, showed a similar low level of viral replication to CK/F98 in infected animal and consequently remained low in pathogenicity. That CK/F98-4664Int virus grew better than CK/F98 virus in cultured MDCK cells further indicated that the replication difference could be attributed at least in part to direct functioning of the internal gene constellation. This notion was further evidenced by the higher activity of H7N9-derived vRNP when compared to that of H9N2-derived vRNP in minigenome assay. Thus, the subunits of viral polymerase, PA, PB1, and PB2, along with NP, are strong candidates for virulence determinants distinguishing H7N9 from H9N2. However, we cannot exclude the candidacy of the other two internal gene-encoding segments, the NS and M segments, which respectively produced two viral proteins, namely NS1 and NS2 and M1 and M2, by alternative splicing. Indeed, previous studies have suggested the involvement of NS and M genes in the emergence of reassortants between H9N2 and H5N1 viruses. NS1 protein, which is only expressed in infected cells, is particularly of interest, given its critical role in evading host innate immunity. Thus, it will be important for future studies to pinpoint which component or combination of internal genes from H7N9 is responsible for the conversion of H9N2 to a mammalian-adapted virus on the advent of H7N9-H9N2 reassortment.

In summary, our study demonstrated the genetic compatibility between human-origin H7N9 and avian-origin H9N2 virus and revealed gain of the internal gene cassette from human H7N9 virus by reassortment as a potential path by which for avian H9N2 to evolve to become a causative agent in mammals. This is in agreement with the concept that the zoonotic potential of an influenza virus is determined by two critical factors: the entry and spread capability associated with HA and cellular permissiveness dictated by internal gene products. A future comparison between CK/F98-4664Int and parental 4664 virus will be needed to validate that the internal gene cassette is fully responsible for the difference between the replication capabilities of H9N2 CK/F98 and H7N9 SH/4664 strains in mammalian cells. It will also be of great interest to determine the detailed mechanism by which gene acquirement shapes the virulence of H9N2 virus. We propose that it is important to closely monitor for the emergence of H9N2 reassortant virus containing a human-derived H7N9 internal gene cassette, which might pose a serious threat to human health.

## Data Availability Statement

The raw data supporting the conclusions of this article will be made available by the authors, without undue reservation.

## Ethics Statement

The animal study was reviewed and approved by Shanghai Public Health Clinical Center.

## Author Contributions

MZ, JX, XZ, and ZL designed the experiments, analyzed the data, and wrote the manuscript. MZ, HC, QT, CZ, SY, LJ, XL, and DF performed the experiments. All authors reviewed and revised the first and final drafts of this manuscript.

## Conflict of Interest

The authors declare that the research was conducted in the absence of any commercial or financial relationships that could be construed as a potential conflict of interest.

## Footnotes

1 www.who.int/influenza
